# Short and Long-Term Trainability in Older Adults: Training and Detraining Following Two Years of Multicomponent Cognitive—Physical Exercise Training

**DOI:** 10.3390/ijerph17165984

**Published:** 2020-08-18

**Authors:** Cristina Blasco-Lafarga, Ana Cordellat, Anabel Forte, Ainoa Roldán, Pablo Monteagudo

**Affiliations:** 1 Physical Education and Sports Department, University of Valencia, 46010 Valencia, Spain; m.cristina.blasco@uv.es (C.B.-L.); ainoa.roldan@uv.es (A.R.); 2 Sport Performance & Physical Fitness Research Group (UIRFIDE), University of Valencia, 46010 Valencia, Spain; pmonteag@uji.es; 3 Statistics and Operational Research Department, University of Valencia, 46100 Burjassot, Valencia, Spain; anabel.forte@uv.es; 4 Education and Specific Didactics Department, Jaume I University, 12071 Castellon, Spain

**Keywords:** agility, cardiovascular fitness, dual tasking, executive function, physical exercise, strength

## Abstract

Despite the benefits of multicomponent physical–cognitive training programs (MC^Cog^TPs), lower training intensities in the concurrent approach, and bigger heterogeneity with aging, suggest the need for long-term analyses, with special attention to training and detraining in older adults. The present study aims to examine these training/detraining effects in a two year MC^Cog^TP, looking for specific dynamics in the trainability of their physical and cognitive capacities. The intervention was divided into four periods: T1, T2 (8 months of training each), and D1, D2 (3.5 months of detraining plus 0.5 of testing each). Twenty-five healthy seniors (70.82 ± 5.18 years) comprised the final sample and were assessed for cardiovascular fitness (6-minutes walking test), lower-limbs strength (30-seconds chair-stand test) and agility (8-feet timed up-and-go test). Inhibition (Stroop test) was considered for executive function. Physical and cognitive status improved significantly (*p* < 0.05) throughout the two years, with larger enhancements for physical function (mainly strength and agility). Strength and cardiovascular fitness were more sensitive to detraining, whilst agility proved to have larger training retentions. Inhibition followed an initial similar trend, but it was the only variable to improve along D2 (d = 0.52), and changes were not significant within periods. Notwithstanding aging, and the exercise cessation in D2, physical and cognitive status remained enhanced two years later compared to baseline, except for lower-limb strength. According to these results, basic physical capacities are very sensitive to training/detraining, deserving continuous attention (especially strength). Both reducing detraining periods and complementary resistance training should be considered. Additionally, physical enhancements following MC^cog^TPs may help cognition maintenance during detraining.

## 1. Introduction

Aging is an irreversible, multifactorial and stochastic process [1,2] where not necessarily, nor homogeneously, the capabilities and integrative adaptive responses of the older adults [3] deteriorate [4,5]. This is due, but not limited, to impaired metabolic, cardiovascular and endocrine functions [2,5,6,7]; structural, physiological and functional changes in the musculoskeletal system [8,9,10]; the worsening of the coupling of fascial tissue, skeletal muscle and nerves [11] and altered neural regulation, with autonomic dysfunction [1,4,6,12] and reduced immune and anti-inflammatory responses [2,5,13]. This comprehensive deterioration results in the worsening of physical and mental conditions, functionality and quality of life [4,9], leading to the vicious cycle of immobilism and aging. It also gives increasing importance to interventions based on physical exercise, widely recognized as the best way to carry out healthy aging [9,14,15,16,17,18] as well as preserve cognitive abilities [9,19,20,21], mainly over the age of 65.

In this scenario, supervised physical exercise training programs (PETPs), combined with complementary medical care, are accepted as powerful health treatments with positive effects on most of the seniors’ own pathologies [15,16,17,22]. Current research is focused on which are the best PETPs (e.g., their adherence or effect-sizes) to improve both, physical [23] and cognitive functions [24]. Lower training attendance with aging accelerates the already mentioned vicious cycle, leading to functional impairment and dependence [25]. It is also important to further understand the long-term effects and level of retention after exercise cessation of PETPs, a concern that has led to an increasing number of publications related to detraining.

On the one hand, neuromuscular approaches, including resistance training [26], coordination [23], or their combination (i.e., multimodal or multicomponent training programs; MCTPs) [27], have been confirmed to enhance the seniors’ physical capabilities [23,28], reducing falls and preventing disability, frailty, morbidity and even death [28]. Bouaziz et al. [27] concluded that MCTPs (endurance, strength, flexibility and balance) were the most effective to improve physical function, cognitive capacities and quality of life. The systematic review by Hortobágyi et al. [23] reported a similar effect size for MCTPs on the self-selected gait, compared to resistance and coordination training (0.86 vs. 0.84 and 0.76, respectively). On the other hand, some recent studies [19,29,30] highlight that physical–cognitive interventions like dancing [30], exergames [31,32], or the addition of cognitive demands into physical exercise in the so called multicomponent cognitive training programs (MC^cog^TPs) [30], are even better strategies to improve both, physical and cognitive outcomes, compared to training programs with an isolated physical capacity.

Notwithstanding, there is still scarce research and a lack of agreement with regard to the deconditioning effects following the cessation of exercise in any multimodal intervention, whether MCTPs or MC^cog^TPs. For example, agility losses have shown to be similar to the strength ones (15% vs. 13%) in a group of senior women undergoing nine months of MCTP, followed by three months of detraining [33], whilst strength decreased more than agility (16% vs. 8%, respectively) in a similar MCTP with an analogous sample [34]. Moreover, some doubts arise in regards to the long-term retention of these complex PETPs, mainly for MC^cog^TPs, since lower intensities and reduced volumes in a particular stimulus, compared to current guidelines [28], might compromise the physical condition maintenance in detraining, for example in strength.

Of uttermost relevance, is the fact that the retention of physical fitness in detraining might contribute to reducing the previously mentioned inflammation, immunosenescence, neuroendocrine dysfunctions and co-morbidities associated with deconditioning, sedentary behaviours and aging [9,15,16,28,35]. It might also help to delay the impairment in the cognitive status [9,36,37]. Therefore, programming exercise interventions looking for stable gains which resist the negative consequences of exercise cessation, should be a key focus when designing any PETP, aiming to optimize its cost-effectiveness and health impact. A long-term impact is crucial because older people have various commitments (e.g., medical and family demands) that make high rates of adherence difficult [38].

In the last decade, our group of research has been working on a MC^cog^TP, registered in 2016 as EFAM-UV© [39] (Spanish acronym for Entrenamiento Funcional para Adultos Mayores property of the University of Valencia). This multicomponent neuromotor exercise training methodology has shown improvements in different populations of older adults [40,41,42,43,44,45,46,47], but it remains unknown its long-term benefits (i.e., the addition of consecutive years of training), as well as the detraining effects following the exercise cessation in summer holiday, both of them purposes of this study. Up to our knowledge, little is known about the long-term effects of MC^cog^TPs in the older adults, most of them lasting < 1 year. Seniors’ trainability (referred to as how they respond to practice) has neither been analysed, looking for specific dynamics in the neuromuscular, metabolic or cognitive long-term changes. Similarly, conclusions with regard to their detraining consequences are also scarce, due to disparity of the research designs because studies vary in training and detraining duration, and few of them analyse changes in physical and cognitive function beyond one year of MC^cog^TPs [34,48].

Therefore, the present study aims to analyse and compare changes in cardiovascular fitness, neuromuscular capacities (strength and agility) and cognitive function (inhibition) in a group of healthy seniors following two years of EFAM-UV© [39], a MC^cog^TP focussing on executive function, postural control and gait training. It is worthy to further understand the dynamics of older adults’ trainability (i.e., training and detraining effects on each capacity) as well as their long-term retention. We hypothesize that both, benefits and losses, would be greater during the first year. Benefits would be larger in the first training period due to the novelty of the program and the younger age of the participants. Losses would be also larger because of less time of exposure to the EFAM-UV© stimuli in this first year.

## 2. Materials and Methods

### 2.1. Experimental Approach

**Figure 1:**
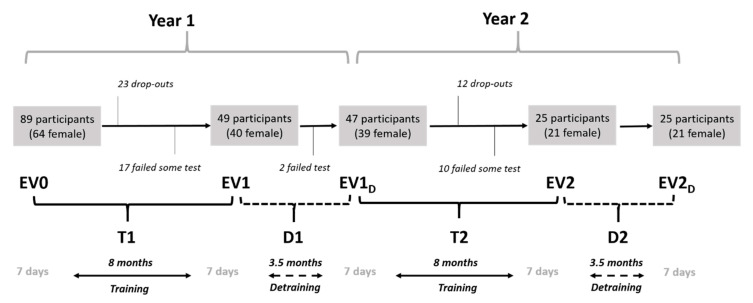
Experimental procedure and flow chart. T1 and T2: first and second training periods (8 months each). D1 and D2: first and second detraining periods (3.5 months each). EV0, EV1, EV1D, EV2, EV2D: pre-post training and detraining assessments (one week each). Upper lines and call texts describe the participants flow, from the initial 89 to the final 25 who completed the whole testing along the two years (from EV0 to EV2D).

In order to look for any specific dynamics in trainability, physical and cognitive capacities were assessed every year at the beginning and the end of the EFAM-UV© exercise intervention, resulting in 8 and 3.5 months for training and detraining, plus 0.5 months for pre-post testing sessions (last week of September and the first week of June every year, respectively) (Figure 1). Within-changes in the five sampling conditions (EV0, EV1, EV1D, EV2 and EV2D) allowed the researchers to analyse short-term changes through two training and two detraining periods: T1, T2 & D1, D2, respectively (Figure 1). Overall training effects (OV-T: from EV0 to EV2), and the final impact after two years of intervention, including the exercise cessation in D2 (INT-2y: from EV0 to EV2D), were considered for long-term analyses.

The training period stopped only for one week at Christmas and one week at Easter every year. Finally, the six-minute walking test (6MWT), the 30s-chair stand test (CST) and the 8-feet timed up-and-go test (TUGT) were included to analyse changes in physical function (cardiovascular fitness, lower limb strength and agility, respectively), whilst the inhibition capacity (Stroop Test-C) was considered for the cognitive status along the two years.

### 2.2. Participants

Inclusion criteria were: being over 65 years old; not suffering an explicit contraindication to the practice of physical exercise; attendance to at least 70% of the sessions and the completion of two consecutive courses, then starting the third one. Exclusion criteria included missing a testing session, becoming ill, or irregular attendance. All the participants, who voluntarily attended the program due to the contact with a specialized care centre for older adults, received extensive information and gave their written consent for this study, approved by the ethics committee of the University of Valencia (H1363126067752). As shown by Figure 1, eighty-nine healthy older adults, who were users of the EFAM-UV© program [39], volunteered to participate. Forty-nine attended regularly and completed the training and testing sessions in T1. Forty-seven of them came back in T2, although only 25 completed the whole battery of assessments over the two years. These 25 came back in the beginning of their third course (EV2D) and constitute the final sample (70.82 ± 5.18 years; 21 female).

### 2.3. Procedures and Performance Outcomes

All the assessments were conducted on two non-consecutive days. Body composition, executive function, agility and strength (in this order) were obtained during the first testing day, while arterial blood pressure and cardiovascular fitness were assessed 48 h later on a second day to avoid interferences.

Height was measured with a stadimeter (SECA 222) and body composition was evaluated by bioimpedance (TANITA, model BC-545N, Tokyo, Japan), on fasting conditions. After body composition, participants sat down and blood pressure was measured twice on the left arm with the arm tensiometer Omron M3 Intellisense (HEM-7051-E) (Omron Healthcare, Kyoto, Japan). In addition, we retained the mean values of systolic and diastolic blood pressure (SBP, DBP) to characterize the sample.

According to Rikli and Jones’ [49] guidelines, physical function testing included the 6MWT, the CST and the TUGT to assess cardiovascular fitness, lower limb strength and agility, respectively. The best of 2 TUGT trials—which measures the time taken by a participant to stand up, walk a distance of 2.44 m, turn around a cone and walk back to the chair and sit down—were considered for agility. Meanwhile the CST, recorded as the maximum number of repetitions of sitting to standing reached during 30 s, and the 6MWT, were performed only once. In order to get their best in this latter one, participants were encouraged to walk as quickly as possible following a rectangular circuit of 20 m long and 5-m wide, with signalling cones every five meters. They were advised of the remaining time once after one minute [49], and the total distance in meters was retained for further analysis. Finally, executive function (inhibition) was assessed through the Stroop test in the Comalli version [50]. The score obtained after 45 s was registered on each sheet, although only sheet C (interference) was retained to analyse the inhibition capacity.

### 2.4. MC^cog^TP Intervention

**Figure 2:**
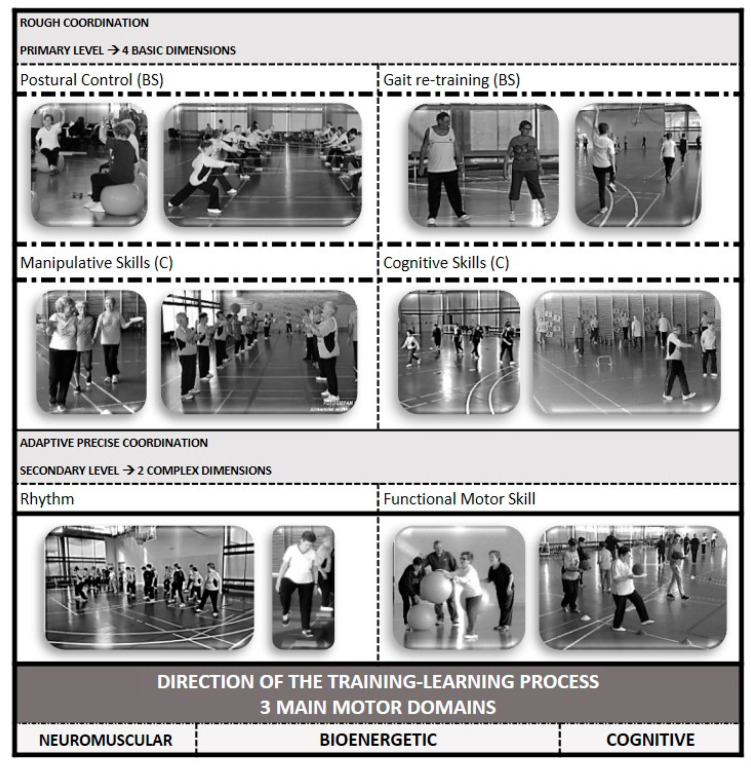
Six dimensions in the older adults’ functional-training psychomotor taxonomy in EFAM-UV©. The six dimensions are distributed in two levels (based on Schnabel, 1994, in Platonov [51]). Any task in the first level aims to improve at least one basic skill (BS: Postural control and/or Gait re-training), combined or not with other basic or complementary skills (C: Manipulative and/or Cognitive Skills). Once the technical improvements allow for individuals to move on with security, the methodology EFAM-UV© introduces new simple exercises but now from its advanced or secondary level. Two more complex dimensions in this level (R: Rhythm and FMS: Functional Motor Skills) help to achieve functional, adaptive, precise coordination. Different tasks in the two levels then progress in complexity and intensity, increasing in demands for any of the three main motor domains (from neuromuscular, to cognitive and/or metabolic orientation), according to a periodized model (Figure 3) EFAM-UV© evolves through the physical–exercise continuum, from strength (neuromuscular contents) to bioenergetics, with cognition as a permanent target. The program is tailored and periodized, increasing and decreasing the psychophysiological load with regards to time of training, and participants’ capabilities and expertise.

The EFAM-UV© program [39] is a MC^cog^TP aiming to improve cognitive, neuromuscular and cardiovascular capacities under a complex approach. On the basis of gait-retraining and improving postural control, enriched environments and continuous changes in constraints facilitate combining strength and cardiovascular proposals under the dual-tasking methodology, increasing intensities and task difficulty, concurrently through the three main motor domains (Figure 2) [39]. In order to increase functionality, this MC^cog^TP includes many tasks related to daily living activities (sitting to standing, asymmetric handling and transporting of light-weights, etc.).

Importantly, the program, which is supervised and conducted by graduates in sport sciences, takes only 60 min per session, twice a week, and it has previously shown improvements in body composition [40], physical function [40,42,43,45,52,53], and executive function [43,52].

**Figure 3:**
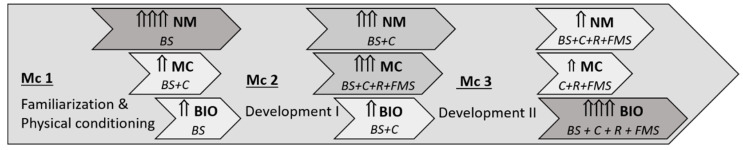
EFAM-UV© periodization. Guidelines of the 3 main mesocycles (Mc) in the 8-months macrocycles used in this study. Horizontal arrows represent the changes in the motor domain and contents’ orientation (NM: neuromuscular; MC: motor control; BIO: bioenergetic), with the length pointing out its beginning and end during the mesocycle. It includes the dimensions to develop in each of them (BS: basic skills; C: complementary skills; R: rhythm; FMS: functional motor skills). The vertical small arrows (and colours) reflect the prevalence and importance of each motor domain.

More specifically, EFAM-UV© sessions start with a 10 to 15 min neuromuscular activation, based on gait training, plus postural control exercises (motor coordination dual-tasks), often combined with cognitive constraints to increase the demands on executive function. Then, there are approximately 15–20 min of strength plus balance exercises (coordinative highly-demanding exercises with elastic bands and dumbbells on alternating days), followed by 15–20 min of bioenergetics (by means of rhythmic exercises or functional motor skills), again on different days and depending on the periodized objectives (from strength to stamina targets, Figure 3). Instead of repeating sets or exercises, EFAM-UV© combines up to 10–12 different polyarticular strength and motor-control exercises in each session (increasing the number of repetitions from 8 to 12, and, on many days, varying the muscle contraction velocities). Exercises are mainly asymmetric, core-demanding, and with wide range of motion and, as soon as possible, they are performed in unstable and/or dynamic conditions. In addition, the progressions are set up through physical conditioning conceptual maps, based on the participants capabilities with regard to the EFAM-UV© taxonomy. This allows to end the macrocycle with gait-training exercises using elastic bands or dumbbells, looking for functional strength and balance while walking, which ensures adaptive executive function demands. According to Blasco-Lafarga, et al. [39], this last mesocycle in EFAM-UV© also includes concurrent metabolic and neuromuscular demands by means of circuit-training and high intensity interval training proposals where the cognitive demand is adapted (most times it is reduced) to the individuals’ capacities. The session ends with a 5 to 10 minute cool down, including amusing and social tasks. As above mentioned, cognitive and physical load is always individually tailored to the participants, following the basis of the EFAM-UV© periodization (Figure 3) [39].

### 2.5. Statistical Analyses

Statistical analyses were conducted using SPSS v.23 (SPSS Inc., Chicago, Illinois, USA). After testing for normality (Shapiro–Wilk test), a Repeated Measures ANOVA test, followed by the Bonferroni post hoc adjustments test, were conducted to compare changes following training (T1, T2) and detraining (D1, D2). Overall training effects (OV-T) and the total intervention (INT-2y) were considered for long-term impact. Later on, in order to homogenise and analyse these changes, the effect size was calculated by means of the Cohen’s d, where the effect was considered small (d = 0.20–0.40), moderate (d = 0.50–0.70) or large (d = 0.80–2.0). The alpha was set at *p* ≤ 0.05.

## 3. Results

Mean of attendance was 70.6% and 71.68% for the first and second year, respectively. Although the aim of the study was to analyse physical and cognitive trainability, Table 1 shows that fat mass and blood pressure diminished significantly (*p* < 0.05), with a small and moderate effect for diastolic and systolic blood pressure, respectively.

Similarly, the within-subjects analyses in the Repeated Measures ANOVA (Table 2) confirmed the overall effect of training/detraining dynamics in all the main variables of the study (*p* < 0.005). This effect was moderate and equal for the three physical outcomes, and a bit lower for the cognition (i.e., Inhibition).

**Figure 4:**
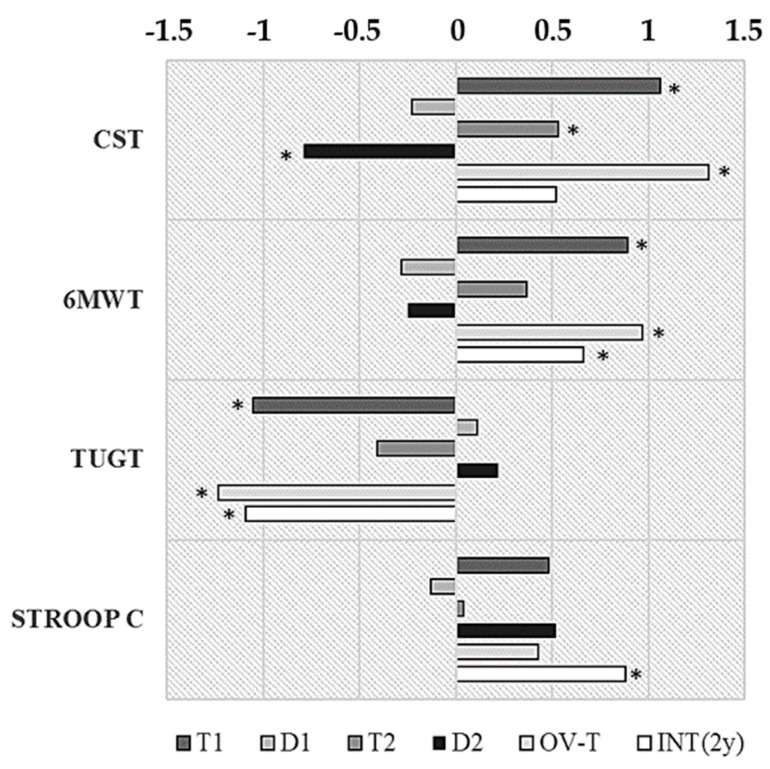
Effect size for the training and detraining changes along two years in EFAM-UV©. T1: changes following the first *8* months of training (EV0 vs. EV1); D1: changes after 3.5 months of detraining (EV1 vs. EV1D); T2: changes following the second 8 months of training (EV1D vs. EV2); D2: changes after the second 3.5 months of detraining (EV2 vs. EV2D). OV-T: Overall changes after two years of training (EV0 vs. EV2); INT-2y: Changes over the two years of intervention, including two training and two detraining periods (EV0 vs. EV2D). * *p* < 0.05. CST: chair stand test, repetitions; TUGT: time up-and-go test, seconds; 6MWT: 6 minute-walking test, meters; STROOPT: stroop test, arbitrary units.

Specifically, shown in Figure 4 and Table 3, all the physical outcomes improved largely in T1 (EV0 vs. EV1 changes in 6MWT, CST and TUG test: *p* < 0.05; d > 0.80), but the cognitive one did not (Stroop test-C: *p* > 0.05; d = 0.48). These beneficial effects on physical function were reduced in T2, with a moderate effect size, and were significant only for CST (EV1D vs. EV2 in CST, *p* = 0.002; d = 0.53). In fact, lower-limb strength always reflected the largest sensitivity to training and detraining. CST was the only test to show a significant and moderate improvement in T2, and an almost large impairment in D2 (EV2–EV2D: *p* = 0.001; d = 0.79), together with the largest overall training effect size (OV-T: *p* = 0.001; d = 1.31 in the comparison EV0–EV2).

The large increase showed by Agility (TUGT) in T1, similar to strength, was followed by non-significant smaller variations over the two years, so detraining effects may have had little contribution to the large overall effect of training in this complex neuromuscular capacity (OV-T: *p* = 0.003; d = 1.23). In fact, agility displayed the largest benefits after two years despite the cessation of exercise in D2 (INT-2y: *p* = 0.001; d = 1.09 as compared to EV0).

Cardiovascular fitness followed similar dynamics, but changes were significant only after T1 (EV0 vs. EV1) and effect sizes were lower compared to neuromuscular capacities (Figure 4 and Table 3). Detraining, slightly larger in the first exercise cessation (D1: d = 0.28; D2: d = 0.25) was not significant, so the outcomes of the 6MWT along the intervention were always better compared to the baseline (EV0).

With regard to cognition, Inhibition only showed a significant improvement and a large effect size when the two years of intervention were considered, which included the months of exercise cessation in D2 (INT-2y: *p* = 0.05; d = 0.88). The percentages of change were similar to physical function over the first year, but they were not significant. Conversely, this variable improved moderately along D2 (d = 0.52).

To Summarize, it is noteworthy that, notwithstanding of aging and the previous months of detraining (D2), the values from the last sampling condition (EV2D) were significantly better than those at EV0 except for CST, which remained only slightly over the starting levels (Figure 4). Considering the short-term training effects, lower limb strength showed the largest percentage of change (23% in T1) and cardiovascular capacity the lowest (12%, also in T1). Considering the whole intervention in the long-term (training plus detraining in the two years, INT-2y), executive function showed the largest benefits (35%) whilst cardiovascular capacity retained few benefits (10%) (Table 3).

## 4. Discussion

As a main finding, physical function is more sensitive to training and detraining than executive function (i.e., inhibition) in our population of healthy older adults following a MC^cog^TP. Strength and cardiovascular fitness reflect bigger significant short-term oscillations over the two years, and more specifically, the dynamics of strength trainability suggest the need for continuous training and/or the reduction in detraining periods in the seniors following MC^cog^TPs. Additionally, profuse and specific agility training in this PET, as well as the complex nature of this neuromuscular capacity, explains agility’s large benefits and good long-term retention. Conversely, inhibition (i.e., executive function) improves slower with training, but decreases little, or even improves, along the detraining periods, underpinning the idea of a holistic positive effect of MC^cog^TPs in healthy older adults. A larger heterogeneity with regard to their cognitive trainability, may also account for this specificity.

As a second finding, training effects were larger in the first year, whatever the outcome and may be due to the first adaptations to new stimuli, as well as to the younger age of the participants, as previously hypothesized. Notwithstanding, with regard to detraining, the worsening of the lower limb strength was higher during the second year, and executive function did not diminish, which was the opposite to our first hypothesis.

Finally, while aging promotes a decline in cardiovascular capacity and lower body strength [9,18,28,54,55], the progressive increase in the mean in every variable and its maintenance above the EV0 throughout the two years of EFAM-UV©, points to this being a well-designed intervention and a proper training stimulus [56]. The exercise cessation in the summer holidays and its short time requirement (60 min, twice a week) were not an inconvenience to attaining the expected mixed moderate effects on physical and cognitive function—a bit larger for the first one— similar to those pointed in a recent review [20].

In terms of physical function, after the expected first large enhancement, with bigger short-term improvements in the two neuromuscular capacities in the study (strength and agility, in this order), participants continued improving during the second year, despite having already displayed a good level of independence, according to Rikli and Jones [57]. Physical fitness improvements might have been somehow more difficult in the second year, because similar stimuli become less demanding the better the physical fitness of the participants. Additionally, this could be because of the attenuation of the learning effect, mainly in the more complex tasks (i.e., agility). The neuromuscular nature of EFAM-UV© was reflected by smaller short-term improvements in the cardiovascular fitness (6MWT), than what was otherwise expected. Notwithstanding, the periodization in EFAM-UV©, and the accumulative effect of training on the long-term, still ensured a large and significant better cardiovascular capacity as compared to baseline (EV0), with a good retention even 14 weeks later. This is of paramount importance in this population, since the cardiovascular system is specially impaired with aging [6,55,58]. Blood pressure and fat mass were also improved in the long term, confirming these beneficial effects and supporting the contribution of MC^cog^TPs (i.e., EFAM-UV©) to neuroendocrine enhanced responses and better cardiovascular health [35,55], in line with aerobic interventions [40,55], and other EFAM-UV© studies [40,42].

As above mentioned, the training/detraining effects were more pronounced in strength, compared to agility. Importantly, losses in this complex capacity, which predicts independence, were small during the summer holidays, and more homogeneous (lower coefficient of variation in %), mostly from the second year of training. This aligns with the study of Leitão, et al. [34], who also integrated strength, balance agility and coordination, thus reducing the time and load of training with regard to resistance training. However, Oliveira, et al. [33] focused their concurrent intervention in resistance training plus aerobic training, and found the opposite results. Similarly, Coetsee and Terblanche [56] found a big loss in agility after 16 weeks of detraining in a program based on resistance training, whilst the worsening in this capacity was not significant after 14 weeks of detraining in EFAM-UV©. These data confirm that the specificity of training matters, whatever the age. Our sample trained for twice the length of time than participants in Coetsee and Terblanche’s study [56] (eight vs. four months), but the neuromuscular complexity and cognitive aspects underlying many tasks in our MC^cog^TP might have also contributed to this improvement and better retention of agility as compared to that after resistance training, as previously suggested [59]. Our study points out specific long-term trainability for agility, with large training retention (i.e., little detraining effects) for those older adults undergoing MC^cog^TPs.

With regard to strength, concurrent physical–cognitive training and the use of dual-tasks, as well as the re-educative approach of EFAM-UV©, reduces the metabolic/endocrine demands as compared to traditional resistance training [20,28], maybe leading to quicker and larger detraining in this capacity, despite its large benefits. The recent position stood by Fragala, et al. [28] summarizes the wide benefits from resistance training on mobility and functional capacity, helping to preserve independence and prevent falls, frailty, disability or event comorbidity and death, over multimodal strategies. A better osteo-muscular and neuroendocrine health associated to the increase in muscle mass (hypertrophy) and the larger neuromuscular intensities in the resistance training [28], might also highlight this need in seniors, following current recommendations [28]. In fact, resistance training for 30 min at least three times per week, maintains the benefits from strength training for almost 27 months before returning to baseline or below [60]. Noteworthy, our results shed new light into the training dependence of strength in older people and suggest the need to shorten the exercise cessation periods after MC^cog^TPs, or the appropriateness of introducing some complementary resistance training and rate of force development exercises in these programs.

With regard to cognition, despite the accepted assumption of improvements in physical fitness as mediators of benefits in cognitive function [19,61], not any exercise intervention ensures significant improvements [20,61]. Dual-tasking, motor control and functional training in MC^cog^TPs help to achieve these enhancements [29,61,62], similar to, although at a smaller level, that of resistance training or aerobic training alone [20,61]. In this sense, our improvements were not significant within periods and we had to wait until the end of the intervention in the second year to see important and significant benefits. The expected large heterogeneity in the inhibition responses to training [63] which was reflected by the largest coefficient of variation in our sample, could be potentially responsible, but our results also reinforce the hypothesis that higher levels of exertion are needed in order to influence the physiological mechanisms improving cognition in the short-term (i.e., brain plasticity and changes at the molecular level [9,20]). Resistance training increases the IGF-1 (**insulin-like growth factor**) hormone signalling and stimulates hippocampal neurogenesis whilst aerobic training influences the production of the neurotrophic factor BDNF (**brain derived neurotrophic factor**), also with the consequence of neurogenesis, as previously summarized [20]. Since EFAM-UV© is a neuromotor training method which first builds up technical factors under the dual-task approach (i.e., postural control, gait retraining and basic skills), and later evolves to higher intensities and metabolic demands, our initial physiological exertion (first mesocycles/first year) might be reduced compared to aerobic and resistance training alone, performed at a certain intensity.

Executive function thus reflects a delayed response compared to the neuromuscular capacities (strength and agility), which are the most sensitive to training in our intervention. The recent review and meta-analysis by Sanders, et al. [24], already points out that executive function improves little in healthy seniors without cognitive impairment, compared to functional improvements (small effect size; Cohen d = 0.27), independently of the exercise dose-parameters (i.e., volume, intensity and frequency). Sanders, et al. [24] do not relate cognitive effects to the program duration, but it is possible that two years of training and more active behaviour during detraining may underpin larger cognitive benefits, after EFAM-UV© (effect sizes increasing from 0.5 to 0.88 in T1 and INT-2y, respectively, once three months of the second detraining had passed). Being physically active has already been shown to reduce cognitive decline [9,61,62,64,65]. Furthermore, healthier lifestyles in people who are aware of the benefits of being active, increase their possibilities to preserve cognitive function [9,66]; a moderator effect that is added to the above cited mediator effect of regular exercise on the executive function [19,20]. Little detraining and long-term significant benefits in our study also support that enhancing mobility and independence in MCcogTPs contributes to keeping a healthy cognitive status in seniors, despite detraining. Indeed, only one day per week along a two year MC^cog^TP has been able to maintain cognition in a similar sample of older adults, whilst their control partners worsened (assessed by means of the Modified Mini-Mental State and the Trail Making Test) [62]. Of uttermost relevance, the methodology supporting EFAM-UV© is designed and periodized to increase the cognitive demands in a similar way to the neuromuscular or cardiovascular demands. Looking forward, it is of paramount important to keep in mind tailoring the difficulty of progression to the individuals’ cognitive and physical thresholds.

Focusing now in the specificity of cognitive detraining, Coelho Júnior, et al. [67] observed that after six months of resistance training, the improvements on executive function disappeared just after one month of detraining in a sample of senior women. It is true that in a previous study [68], executive function also returned to baseline after three months of detraining following a MC^cog^TP, but the sample was older, executive function was assessed with the Mini-Mental State Examination and the intervention may have been too short. Losses in cognition detraining have already been shown to be quicker the older the participant [20,69], so we urge early interventions.

On the other hand, while some authors establish a range between 69.1% and 75% of adherence to get physical exercise benefits in long-term interventions [25], others suggest the need of surpassing the 85% to improve memory or executive function [65]. The mean attendance of those completing the whole battery of tests in this two year EFAM-UV© intervention reduced to 70.6% and 71.68% (first and second year, respectively). Differences in the physical and cognitive trainability in our study align with this need for larger adherences, or longer periods of training to ensure long-term physical and cognitive improvements,

To conclude, we had hypothesized larger changes in both cognitive and physical functions following the first year of intervention (including training and detraining), due to the novelty of stimuli and the younger age of the participants. Effectively, the first training effects confirmed to be larger, similarly to Nordgren et al. [70]. However, we failed with regard to detraining, where the effects were larger for strength in the second year. The age of our sample could be a cause of this, counteracting the longer duration of the stimuli following two years of training, since Leitão, et al. [34] found a larger loss in their first detraining with a younger sample (a mean age of 70 vs. 66 years, respectively). Again, comparisons within studies are difficult, and opposite to our data, their first detraining displayed a severe decline (16.01% in Leitão et al. [34] compared to 4.54% in our sample). Losses were similar during the second detraining (13.92% vs. 15.7%). Every year is therefore important in these later stages, confirming the quicker loses with age [69]. Increasing the total weekly exercise duration to meet the guidelines for older people [71] might also help, despite the large benefits following this two day (120 min weekly) training program.

Summarizing, significant benefits and impairments from both neuromuscular and cardiovascular capacities over the two years of our study, and their mediator and moderator effects on cognition [19], point out the need to minimize detraining periods with aging. Indeed, the introduction of little training pills have already been suggested [60], in order to maximize the training effects and to preserve a higher physical functionality and greater autonomy during detraining. The addition of some complementary resistance training in MC^cog^TPs is also recommended [28].

As a limitation of the study, the lack of a control group prevents from comparisons with non-active older adults, but we found that it was not ethical to assess any senior sample without offering an alternative intervention during two years, this was considered unacceptable for the researchers. Furthermore, the EFAM-UV© initial studies already proven its benefits, both physical and cognitive, compared to two control groups (only cognitive training, and only traditional gymnastics for seniors) [52]. Additionally, accelerometery or some questionnaires related to daily living activities would have been also a valuable and complementary source of information to explain differences in trainability, mainly those related to changes in detraining. It is noteworthy to clarify that, unfortunately, some older adults failed to attend only a few testing sessions along the two years, so their data were invalid and the sample reduced from the initial 89 to the final 25.

## 5. Conclusions

In the short-term, physical function is more sensitive to training and detraining than executive function in elderlies undergoing MC^cog^TPs. Executive function improves greatly, but needs longer periods of cognitive–physical exercise training. It also enhances despite exercise cessation in physically active older adults.

The largest sensitivity for lower-limb strength highlights its key role and the priority of developing neuromuscular PETPs against immobilism. Our results confirm the importance of strength and cardiovascular training in order to maintain autonomy with aging, and gives a hint to health fitness professionals and personal trainers to program little neuromuscular and cardiovascular pills during any detraining period in order to sustain not only functionality, but also cognitive function. Increasing the neuromuscular or cardiovascular demands, mainly the first, through the addition of some resistance training—specific microcycles interspersed along the MC^cog^TPs macrocycle, or the reduction in the length of detraining periods—might be also considered.

Earlier MC^cog^TPs addressed to younger old people (>60 years) might optimize the benefits and be more cost-effective, both for cognitive and functional status.

## 6. Patents

The multicomponent cognitive training program is an intellectual property from the University of Valencia.

## Figures and Tables

**Table 1:** Body composition and Blood Pressure following two years of EFAM-UV© expressed as mean (*SD*).

Variables	EV0	CV (%)	EV2_D_	CV (%)	*p*	*d*
Weight (kg)	66.02 (10.03)	15.19	65.09 (9.15)	14.06	0.20	−0.10
Height (m)	1.56 (0.07)	4.49	1.55 (0.07)	4.52	0.30	−0.14
Lean Body Mass (kg)	40.67 (7.11)	17.48	41.08 (7.35)	17.9	0.52	0.06
Fat Mass (kg)	34.97 (5.80)	16.59	32.85 (5.56)	16.93	0.02	−0.37
SBP (mmHg)	133.75 (25.11)	18.77	123.14 (12.21)	9.92	0.04	−0.54
DBP (mmHg)	75.79 (11.40)	15.04	71.43 (8.34)	11.68	0.02	−0.44

SBP = systolic blood pressure; DBP = diastolic blood pressure; *d*: Cohen Effect Size; CV: coefficient of variation.

**Table 2:** Training and detraining changes in physical and cognitive function along two years of EFAM-UV©.

Tests of Within-Subjects Effects							
**Intervention**	**Variables**	**Type III Sum of Squares**	**Df**	**Mean Square**	**F**	***p***	**Partial eta Square**
	6MWT	56,455.07	4	14,113.77	14.33	0.001	0.46
	CST	279.62	4	67.65	14.2	0.001	0.44
	TUGT	29.01	4	7.25	16.45	0.001	0.46
	STROOPT	644.43	4	161.11	5.27	0.001	0.32

**Table 3:** Training and detraining changes in physical and cognitive function along two years of EFAM-UV© expressed as mean (*SD*).

Sampling Conditions	CST (*rep*)	CV (%)	TUGT (*s*)	CV (%)	6MWT (*m*)	CV (%)	STROOPT (*a.u*)	CV (%)
EV0	15.47 (3.13) ^a,b,c^	20.23	6.9 (1.54) ^a,b,c,d^	22.32	507.61 (73.8) ^a,b,c,d^	14.54	28.92 (9.22) ^d^	31.88
EV1	19.13 (3.75)	19.6	5.60 (0.83)	14.82	571.86 (70.6)	12.35	34.17 (12.5)	36.58
*∆T1*	*23.65%*		*−18.84%*		*12.65%*		*18.15%*	
EV1_D_	18.26 (3.71) ^c^	20.32	5.68 (0.54)	9.51	551.67 (74.5)	13.5	32.58 (12.32)	37.81
*∆D1*	*−4.54%*		*1.43%*		*−3.53%*		*−4.65%*	
EV2	20.45 (4.43) ^d^	21.66	5.42 (0.72)	13.28	579.17 (74.13)	12.8	33 (9.81)	29.73
*∆T2*	*11.99%*		*−4.58%*		*4.98%*		*1.29%*	
EV2_D_	17.24 (3.61)	20.94	5.58 (0.74)	13.26	559.17 (83.2)	14.88	39.08 (13.43)	34.37
*∆D2*	*−15.7%*		*2.95%*		*−3.45%*		*18.42%*	
*∆ OV-T*	*32.19%*		*−21.45%*		*14.08%*		*14.11%*	
*∆ Int-2y*	*11.44%*		*−19.13%*		*10.16%*		*35.13%*	

^a^ different from EV1; ^b^ different from EV1_D_; ^c^ different from EV2; ^d^ different from EV2_D_; CST: chair stand test, repetitions; TUGT: time up-and-go test, seconds; 6MWT: 6 minute-walking test, meters; STROOPT: stroop test, arbitrary units. CV: coefficient of variation, in %.
